# Comparative Genomic and Functional Analysis of c-di-GMP Metabolism and Regulatory Proteins in *Bacillus velezensis* LQ-3

**DOI:** 10.3390/microorganisms12081724

**Published:** 2024-08-21

**Authors:** Rong Li, Panlei Yang, Hongjuan Zhang, Chunjing Wang, Fang Zhao, Jiehui Liu, Yanbin Wang, Yan Liang, Ting Sun, Xiansheng Xie

**Affiliations:** 1 Institute of Wheat Research, Shanxi Agricultural University, Linfen 041000, China; lirongxms@sxau.edu.cn (R.L.); zhjxms@sxau.edu.cn (H.Z.); m18247678345@163.com (C.W.); zhaofangxms@sxau.edu.cn (F.Z.); liujiehui@sxau.edu.cn (J.L.); wangyanbinxms@sxau.edu.cn (Y.W.); 208020ly@sxau.edu.cn (Y.L.); 18717323157@163.com (T.S.); 2 Department of Plant Pathology, College of Plant Protection, China Agricultural University, Beijing 100193, China; yangpanlei1993@163.com

**Keywords:** *Bacillus velezensis*, c-di-GMP, comparative genomics, biocontrol, wheat sharp eyespot

## Abstract

*Bacillus velezensis* is a promising candidate for biocontrol applications. A common second messenger molecule, bis-(3,5)-cyclic-dimeric-guanosine monophosphate (c-di-GMP), has the ability to regulate a range of physiological functions that impact the effectiveness of biocontrol. However, the status of the c-di-GMP signaling pathway in biocontrol strain LQ-3 remains unknown. Strain LQ-3, which was isolated from wheat rhizosphere soil, has shown effective control of wheat sharp eyespot and has been identified as *B. velezensis* through whole-genome sequencing analyses. In this study, we investigated the intracellular c-di-GMP signaling pathway of LQ-3 and further performed a comparative genomic analysis of LQ-3 and 29 other *B. velezensis* strains. The results revealed the presence of four proteins containing the GGDEF domain, which is the conserved domain for c-di-GMP synthesis enzymes. Additionally, two proteins were identified with the EAL domain, which represents the conserved domain for c-di-GMP degradation enzymes. Furthermore, one protein was found to possess a PilZ domain, indicative of the conserved domain for c-di-GMP receptors in LQ-3. These proteins are called DgcK, DgcP, YybT, YdaK, PdeH, YkuI, and DgrA, respectively; they are distributed in a similar manner across the strains and belong to the signal transduction family. We selected five genes from the aforementioned seven genes for further study, excluding YybT and DgrA. They all play a role in regulating the motility, biofilm formation, and colonization of LQ-3. This study reveals the c-di-GMP signaling pathway associated with biocontrol features in *B. velezensis* LQ-3, providing guidance for the prevention and control of wheat sharp eyespot by LQ-3.

## 1. Introduction

Pests and diseases are a major threat to crop yields in the field of agriculture. To counteract this, chemical pesticides are a popular method of pest and disease management, with the goal of protecting both the quantity and quality of agricultural output. However, the use of chemical pesticides also leads to a series of issues, such as residual chemicals, environmental pollution, and drug resistance [1]. Therefore, developing natural and ecologically friendly biological agricultural agents is of paramount importance. Among these agents, *Bacillus* species have the ability to form environmentally resistant spores and have a long shelf life [2,3]. As a result, there has been a significant rise in the availability of biocontrol agents formulated with *Bacillus* species in the market. For example, *Bacillus amyloliquefaciens* PQ21 effectively controls tobacco black shank and tobacco bacterial wilt disease. Additionally, *Bacillus cereus* is used to control rice neck blast and rice sheath blight, while *Bacillus subtilis* is utilized to control a variety of crop diseases, including wheat, cotton, and rice. These species exert their disease control effects through various mechanisms, including the secretion of antifungal compounds, competing with pathogens for nutrients and survival sites, and inducing host plants to produce disease resistance [3,4]. The use of biological control agents presents certain challenges, the most notable being the unstable efficacy of their application in the field. This instability is primarily caused by environmental factors and the genetic regulation of the strains themselves [5,6,7]. Therefore, studying the regulatory mechanisms of the biocontrol strain is crucial for understanding the biocontrol mechanism.

Cyclic diguanylate (c-di-GMP), a novel second messenger molecule, is widely present in bacteria and was discovered in *Gluconacetobacter xylinus* [8]. C-di-GMP is a small molecule that is synthesized from two molecules of GTP through the action of diguanylate cyclases (DGCs) that contain a GGDEF domain. This molecule is hydrolyzed by specific phosphodiesterases (PDEs) that contain either an EAL or HD-GYP domain, resulting in the formation of linear pGpG. This linear form can then be further degraded into GMP by oligoribonucleases [9,10]. Moreover, these enzymes have various sensor domains, such as N-terminal response regulator receiver (REC), Per/Arnt/Sim (PAS), and cGMP phosphodiesterase/adenylyl cyclase/FhlA (GAF) domains. These domains can modify enzymatic activities in response to external stimuli [11,12,13]. C-di-GMP is recognized by downstream receptors that are associated with specific signal transduction pathways [14]. The classes of c-di-GMP receptors include proteins with PilZ domain, GGDEF/EAL domain proteins that can bind c-di-GMP but are enzymatically inactive, c-di-GMP specific riboswitches, trigger PDEs, and transcriptional regulators [15,16]. The regulation of c-di-GMP concentration in bacteria is typically orchestrated by its synthesizing and degrading enzymes, while the receptor discerns fluctuations in c-di-GMP levels to modulate its physiological activities. Previous reports have concluded that c-di-GMP can regulate various important physiological processes, including motility, biofilm formation, antifungal antibiotic production, and host colonization [17,18]. Overexpression of genes responsible for c-di-GMP synthesis in *Pseudomonas putida* KT244 leads to a decrease in motility and colonization of plant roots [19]. It is currently recognized that the c-di-GMP signaling molecule plays a role in regulating the biocontrol features of *Bacillus* [20]. Mutants lacking the synthesis enzyme *ytrP* or the degradation enzyme *yuxH* significantly reduce biofilm formation and motility, resulting in decreased colonization on cucumber roots for *B. velezensis* SQR9 [21]. Overexpressing *ytrP* in *B. velezensis* PG12 has been shown to stimulate biofilm formation [22]. The diversity of c-di-GMP metabolic enzymes and receptors underscores the intricate nature of the c-di-GMP signaling pathway.

*Bacillus*, as one of the model strains of Gram-positive bacteria, has been the subject of extensive research into its intracellular c-di-GMP signaling pathway. *B. subtilis* contains three DGCs (DgcK, DgcP, and DgcW), one active PDE (PdeH), and three putative c-di-GMP receptors (MotI, YdaK, and YkuI) [23,24,25,26]. Among these, MotI (formerly called DgrA) is a protein that contains a PilZ domain. When the concentration of c-di-GMP increases within the cell, MotI binds to it, causing a conformational change, and then interacts with MotA to inhibit bacterial motility [24,26,27]. As a significant member of the *B. subtilis* branch, *B. velezensis* is a promising candidate for biocontrol applications in agriculture due to its broad antibacterial spectrum and metabolic diversity [28]. However, research on the c-di-GMP signal pathway in this species is still limited. *B. velezensis* SQR9 has been found to contain three proteins with the GGDEF domain (YdaK, YhcK, and YtrP) and two proteins with the EAL domain (YuxH and YkuI). YtrP, YuxH, and YkuI play a role in the colonization of cucumber plant roots [21]. Meanwhile, four proteins with the GGDEF domain (YdaK, YhcK, YtrP, and YybT) and two proteins with the EAL domain (YuxH and YkuI) have been identified in *B. velezensis* PG12. The receptor YdaK affects the formation of biofilms and ultimately affects biocontrol activity [20,22]. Comparative analysis of c-di-GMP metabolic enzymes in SQR9 and PG12 revealed that they have similarities in the type and number of these enzymes, with only YybT differing between the two strains. 

The strain LQ-3 was isolated from wheat rhizosphere soil. This study demonstrated the effectiveness of LQ-3 against wheat sharp eyespot and identified it as a species of *B. velezensis* through genome-wide phylogenetic analysis. Then, we analyzed the distribution of c-di-GMP metabolic and regulatory genes in LQ-3. Additionally, we performed a thorough analysis of the genes implicated in the c-di-GMP signaling pathway in *B. velezensis,* including an examination of the number of genes involved. Moreover, we examined the function of c-di-GMP-metabolism-related genes in regulating biological features in LQ-3. We were able to gain a better understanding of the genetic underpinnings of this important signaling pathway in LQ-3. These results offer a theoretical basis for examining the influence of c-di-GMP on the biocontrol effectiveness of LQ-3.

## 2. Materials and Methods

### 2.1. Strains and Growth Conditions

In this study, we utilized the *B. velezensis* strain LQ-3, which was isolated from wheat soil and has been preserved in our laboratory. This particular strain was chosen for whole-genome sequencing due to its exceptional biocontrol performance. Strain LQ-3 was routinely grown at 37 °C on Luria–Bertani (LB) broth or on solid LB medium supplemented with 1.5% agar. The pathogen *Rhizoctonia cerealis,* which was isolated from a diseased wheat plant, was used as an indicator fungus to detect antifungal activity. The fungal strain was stored in paraffin at 4 °C at the Institute of Wheat Research, Shanxi Agricultural University. The *R. cerealis* pathogen was cultivated on a wheat grain agar plate for propagation in a 25 °C culture chamber for 15 days prior to utilization. The overnight bacterial liquid culture was harvested by centrifugation, and the pellet was diluted to 10^6^, 10^7^, and 10^8^ bacteria suspensions with sterile water.

### 2.2. Antifungal Assay

To test the activity of LQ-3 against the fungal pathogen *R. cerealis*, we conducted a plate confrontation assay. Initially, we activated the pathogen *R. cerealis* and the biocontrol bacterium LQ-3 in potato dextrose agar (PDA) and LB media, respectively, for 5 days and 24 h. Next, we inoculated the pathogen (a 5 mm diameter block of mycelium) in the center of a 90 mm diameter petri dish, while the bacteria were inoculated 20 mm away from the pathogenic fungus, using a volume of 150 μL [29]. Additionally, the Oxford cup method was used following the same procedure as described above for the placement of the pathogenic fungus and biocontrol bacteria to evaluate the antagonism activity of LQ-3. Specifically, 150 μL of the overnight shaking culture of LQ-3 was placed in the Oxford cup, while sterile water was used as a blank control. Each treatment was repeated three times and incubated at a constant temperature of 28 °C in a culture box. The width of the inhibition zone was measured when the blank control almost filled the entire petri dish. This measurement was used to calculate the inhibition rate.

To evaluate the efficacy of LQ-3, healthy wheat seeds were selected for germination, and uniformly sprouted seeds with good plumpness were chosen. These seeds were then immersed in different concentrations of bacterial suspensions for 3 h. Following this, ten seeds from each treatment were sown onto PDA plates that had been cultured with *R. cerealis* for 7 days. Vermiculite was used for moisture retention during the culturing process. The experiment involved replicating each treatment three times, with sterile water immersion serving as the control. The plates were then placed in a light incubator set at 20 °C with a 12 h photoperiod and regularly irrigated. After 15 days, the wheat disease index was investigated using the criteria established by Lipps et al. [24]. In the pot experiment, a mixture of sterilized soil and wheat granular sand in a 15:1 weight ratio was created and filled in the nutrient bowl. Germinated wheat seeds were then soaked in 10^7^ concentration bacterial suspension for 3 h. Ten seeds per pot were cultivated in the nutrient bowl, with sterile water immersion as the control. Adequate water was provided, and the disease condition was checked 25 days after planting. The wheat sharp eyespot disease grading criteria are also referred to in a published paper [30].

### 2.3. Genome Sequencing, Assembly, and Annotation

A single colony of *B. velezensis* LQ-3 was incubated and cultured overnight in LB broth at 37 °C. Genomic DNA was extracted using the Bacterial Genomic DNA Extraction kit (Solarbio, Beijing, China), and its quality and quantity were measured using a NanoDrop ONE instrument (Thermo Fisher Scientific, USA). The DNA was subsequently sequenced using an Illumina NovaSeq 6000 and Oxford Nanopore GridION platform by Wuhan Benagen Technology Solutions Com., Ltd (Wuhan, China). In order to perform Illumina sequencing, a 1 μg DNA sample was fragmented using an ultrasonication approach, size-selected, and end-repaired. Each fragment produced was then ligated to an Illumina-specific adapter sequence. The resulting product was quantified, indexed, and sequenced on the NovaSeq 6000 platform. For long-read sequencing, genomic DNA was purified and directly constructed into a library using a ligation sequencing kit (SQK-LSK109, Hangzhou, China) following the manufacturer’s protocol. The DNA library was barcoded using the ONT standard protocol with the native barcoding expansion 1-12 kit. The library was then loaded onto R9.4.1 flow cells and sequenced on a PromethION sequencer (Oxford Nanopore, UK). The company filtered out low-quality reads using scripts. The high-quality short-read and long-read sequences were assembled into a complete sequence using Unicycler v.0.4.9 with default settings [31]. Prokka v1.12 was used to predict protein-coding genes followed by functional annotation of the protein-coding genes using the Basic Local Alignment Search Tool (BLAST) against the COG, Kyoto Encyclopedia of Genes and Genomes (KEGG), and Interpro databases [32,33]. The gene locations, GC skew, and GC content of the final annotated genome sequence were plotted using CIRCOS [34]. Additionally, the antiSMASH v4.0.0rc1 program was utilized to examine the secondary metabolite gene clusters [35].

### 2.4. Phylogenomic Analysis

In order to construct a phylogenetic tree for *B. velezensis* LQ-3 and other *Bacillus* genomes, we downloaded all the genome sequences of *Bacillus* from the National Center for Biotechnology Information (NCBI) database. We included *Paenibacillus polyxyma* M1 as an outgroup. To establish the maximum-likelihood (ML) phylogenetic tree of *Bacillus* species, we used a sequence of single-copy core proteins shared by the *Bacillus* genomes. The single-copy core gene was extracted from the PGAP (Version 6.8) table using our own Perl scripts. Subsequently, multiple alignments of amino acid sequences were constructed using MAFFT v7.310, and conserved blocks from these alignments were selected using Gblocks 0.91b [36]. The maximum likelihood tree was then constructed using the RAxML v8.2.10 software and the PROTGAMMALGX model with 100 bootstrap replicates [37]. The tree was generated using MEGA v7.0.26 software [38]. Additionally, average nucleotide identity (ANI) values between two genome sequences were determined using JSpecies software v1.2 with MUMmer alignment [39]. A heat map was then created using the pretty heatmaps 1.0.12 to confirm the results.

### 2.5. Pan-Genome Analysis

To identify core and specific genes in *B. velezensis* strains, we conducted a pan-genome analysis using the PGAP software (Version 6.8) after genome annotation in Prokka [40]. The Pan-Genome Analysis Pipeline (PGAP)-based protein similarity method was utilized to detect a set of core orthologs from the 30 *B. velezensis* strains. These core orthologs were then clustered based on at least 50% protein sequence identity and 50% overlap with the longest sequence, with an e-value of 1e-5 [33]. To select the core genome and specific genomes from the pan-genome table, we used our own Perl script [37]. The COG database was used for functional annotation of the pan-genomes of *B. velezensis*. We visualized the core genome and pan-genomes using PanGP v1.0.1 software and generated distribution plots of total genes and conserved genes found upon progressive sampling of ‘n’ genomes using default parameters [41].

### 2.6. Involvement of Proteins with Conserved Structural Domains and Quantitative Analysis in the c-di-GMP Metabolism and Regulation

In this study, we used HMMER to identify genes that contain GGDEF, EAL, and PilZ domains from 30 strains of *B. velezensis*. We selected genes with an e-value of 1e-5 or less. Next, we employed TBtools to extract the corresponding protein sequences and analyzed the conserved domains and amino acid sites using the Conserved Domain Database (CDD) and Simple Modular Architecture Research Tool (SMART). Aligned amino acid sequences were obtained through multiple protein sequence alignments using DNAMAN9.0. The resulting conserved motif sequence was then visualized using WebLogo 2.8.2.

### 2.7. Structural Analysis of Proteins Involved in c-di-GMP Metabolism and Regulation

The GGDEF, EAL, and PilZ domains in *B. velezensis* LQ-3 were modeled using SWISS-MODEL (SWISS-MODEL (expasy.org)). Templates were sorted by their expected model quality and assessed using Global Model Quality Estimation and Quaternary Structure Quality Estimate [42,43]. In this study, the target protein sequence was uploaded to SWISS-MODEL for analysis. Various factors were taken into consideration, including the GMQE value of the template, QSQE value, and alignment with the targeted sequence and ligand sites in order to select suitable templates for modeling. Based on these criteria, WspR from *P. aeruginosa* containing the GGDEF domain, RmcA of the GGDEF-EAL hybrid domain, and MotI from *B. subtilis* containing the PilZ domain were chosen as templates. The constructed models were saved in PDB file format. Protein structure alignment was performed using PyMOL software 3.0, and the quality of the alignment was assessed based on the RMSD value. The target PDB files and their corresponding template PDB files were then uploaded to PyMOL software for protein sequence overlap alignment. A smaller RMSD value indicates a higher similarity between the two protein structures.

### 2.8. Construction of Knockout Mutants in B. velezensis LQ-3

According to the whole-genome sequence of *B. velezensis* LQ-3, the putative c-di-GMP metabolic genes in LQ-3 were deleted as described with slight modifications. Fragments of 900 bp upstream and 900 bp downstream of the target gene were amplified from the LQ-3 genome, and the primers used are listed in Table S6. The pMDE vector was digested with *Hind III* and *Spe I*. The source and detailed information of the pMDE vector are referred to in Table S5. All PCR products and the digested pMDE were gel purified using a TIANgel midi purification kit (Tiangen, Taiyuan, China). The vector was linked to the upstream segment of the target gene through a seamless cloning kit, and then the above vector was digested with *BamH I* and *Sac I*. The digested vector was gel purified and subsequently ligated with the downstream fragment of the target gene to obtain the recombinant plasmid vector. The *Bacillus*-*Escherichia coli* shuttle vector pUBXC was introduced into *B. velezensis* LQ-3 through electroporation to obtain the strain LQ-3 (pUBXC). Plasmid pUBXC is formed by connecting the xylose induction system of the pUBX vector with the *comK* fragment, which is related to the formation of competence (Table S5) [44]. A single colony of LQ-3 (pUBXC) was selected and cultured overnight in 5 mL of LB liquid medium at 200 rpm. The seed culture was then inoculated into fresh 5 mL LB liquid medium at a dilution of 1:50 and incubated at 37 °C and 200 rpm until reaching an OD_600_ of 0.5. Xylose at a final concentration of 0.2% was added for induction at 37 °C and 170 rpm for 1 h [44]. The bacterial culture was then aliquoted into 2 mL centrifuge tubes, with 200 μL in each tube, followed by the addition of 10 μL of the corresponding recombinant vector (Table S5). After incubating at 37 °C and 120 rpm for 3 h, the bacterial suspension was spread onto LB agar plates containing zeocine and incubated at 37 °C until single colonies appeared. Genomic DNA extraction and verification of mutants were carried out by PCR using the primers listed in Table S6.

### 2.9. Bacterial Growth Rate Analysis

The test strain was activated on a 37 °C agar plate and then incubated overnight in a shaker. The seed culture was transferred to a fresh medium, and the OD_600_ of the bacterial suspension was measured every 2 h for a total of 12 h.

### 2.10. Swarming Motility Assay

The swarming assay was conducted to measure the multicellular motility of bacteria. Specifically, 3 μL of bacterial culture with an OD_600_ of 0.8 was inoculated at the center of a 0.7% LB agar plate. The swarming plates were then incubated for 6 h at 37 °C, and the diameter of the motility zone was measured. Each assay was carried out with three independent experiments, each consisting of at least three technical replicates per strain.

### 2.11. Biofilm Formation Assay

The development of biofilms can be categorized into the formation of a pellicle on the liquid–gas interface and the growth of biofilm colonies on solid surfaces. Pellicle: Inoculate the target strain into LB medium and incubate it overnight at 200 rpm/min and 37 °C. The following day, transfer it to a fresh LB medium. Prepare Msgg medium in 12-well cell culture plates and inoculate the bacterial liquid of the test strain at a ratio of 1:1000. Once the OD_600_ reaches 0.8, spot it onto Msgg medium using approximately 4 mL of culture medium and 4 μL of bacterial liquid. Incubate them all at 28 °C for three days to observe any morphological changes in the biofilms formed by each strain, with three replicates for each. Colony: Similarly, start by inoculating the target strain into LB medium and cultivating it overnight at 200 rpm/min and 37 °C before transferring it to fresh LB medium. Create a solid plate with a final concentration of 1.5% Msgg by mixing 2X Msgg culture medium with sterilized water agar (3%). When the OD_600_ reaches 0.8, small amounts (4 μL) of bacterial liquid from each strain are spotted onto separate Msgg plates, which are then dried and placed in a 28 °C incubator for three days to observe wrinkling degree and morphology changes in biofilm colonies. As with pellicle formation, three replicates are established for each strain.

### 2.12. Root Colonization Capability Assay

The sterilized wheat seeds were cultured in an incubator until white shoots emerged and were then soaked in the fermentation broth of each strain for 3 h. A control group was soaked in sterile water. Subsequently, the seeds were planted in nutrient pots with sterile soil. Wheat seedlings were examined and tested every 5 days after emergence from day 7. During the test, the roots of the wheat seedlings were first washed, dried, and weighed after cutting. The severed roots were disinfected with 75% ethanol, then rinsed with sterile water 3 to 5 times. After that, they were ground in an equal volume of sterile water and centrifuged to obtain the tissue fluid. The plate dilution method was used for quantification [45]. Tissue fluid was plated on resistant plates, and colonies with similar morphology were counted.

## 3. Results

### 3.1. Biocontrol Efficacy of B. velezensis LQ-3

**Figure 1:**
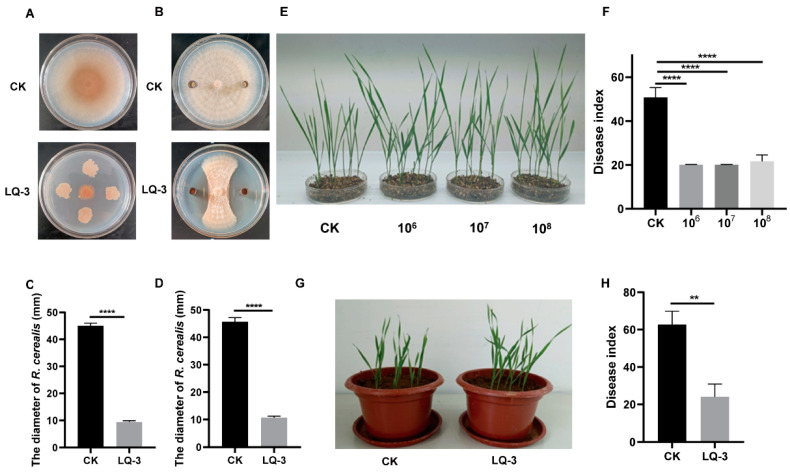
Strain LQ-3 could serve as a potential biocontrol agent against *R. cerealis*. (**A**,**B**) The antagonistic activity of both the strain LQ-3 (**A**) and its fermentation broth (**B**) toward *R. cerealis*. (**C**,**D**) The biocontrol strain LQ-3 (**C**) and fermentation broth (**D**) significantly inhibited the growth of mycelia. (**E**) The biocontrol effect of LQ-3 toward *R. cerealis* under different concentrations (10^6^, 10^7^, and 10^8^ CFU/mL) on the plate. (**F**) The disease index of control and LQ-3 (10^6^, 10^7^, and 10^8^ CFU/mL) in plate experiments. (**G**) The biocontrol effect of LQ-3 (10^7^ CFU/mL) toward *R. cerealis* in a greenhouse experiment. (**H**) The disease index of control and LQ-3 (1 × 10^7^ CFU/mL) in greenhouse experiments. Error bars indicate ± SD of three replicates. The statistical analysis was performed using GraphPad Prism 8 software by *t*-test and one-way ANOVA (*p* < 0.05). ****, *p* < 0.0001, and **, *p* < 0.01).

In previous work, we isolated a total of 65 strains of bacteria from wheat soil samples collected in Shanxi province (36°13′02″ N, 111°33′07″ E). Our results showed that six strains have the potential to control *Rhizoctonia cerealis* (Table S1). After the primary screening, we found that strain LQ-3 exhibited distinctive inhibition of *R. cerealis*. This strain exhibited the ability to inhibit mycelial growth, as evidenced by the presence of a clear inhibition zone. The results revealed that in the PDA medium, the diameter of mycelium in the control group was 45.00 ± 1.00 mm, whereas in the treatment group it was 9.33 ± 0.58 mm (Figure 1A,B). Additionally, it was observed that the fermentation broth of strain LQ-3 effectively prevented the growth of hyphae with a noticeable inhibition zone, indicating that certain secondary metabolites excreted by the LQ-3 fermentation broth may have exerted inhibitory effects on the growth of *R. cerealis* mycelium. The mycelial diameters of the control and fermentation broth groups were 45.67 ± 1.53 mm and 10.67 ± 0.58 mm, respectively (Figure 1C,D). The inhibition rates for both the strain and fermentation broth were 79.33% and 75.56%, respectively. Subsequently, we evaluated the potential of strain LQ-3 to serve as a biological control agent for wheat sharp eyespot under both plate and greenhouse conditions. Various concentrations were tested in the plate experiment. The results indicate that the disease index of the control wheat plants was notably higher than that of the treated wheat. Moreover, there was no significant difference in the biocontrol efficiency for the 1 × 10^6^, 1 × 10^7^, and 1 × 10^8^ CFU/mL concentrations (Figure 1E,F). Based on the findings, the concentration of 1 × 10^7^ CFU/mL was chosen for the follow-up experiments since wheat seedlings treated with this concentration exhibited the best growth. Pot experiments demonstrated that the wheat plant treated with LQ-3 had a lower disease index (24.29%) compared to the control plant treated with water (63.08%) for 25 d. This finding supports the biocontrol effectiveness of LQ-3 against wheat sharp eyespot (Figure 1G,H).

### 3.2. Genome Features of Strain LQ-3

The genome of *B. velezensis* LQ-3 was sequenced using Nanopore and Illumina platform. A total of 2.00 Gbp and 2.97 Gbp of clean data were collected after quality control. The complete genome of LQ-3 comprises a single circular chromosome spanning 3,929,792 bp with an average G + C content of 46.50%. In total, 3861 genes were identified, including 3747 coding sequence genes (CDSs), 86 tRNA, 27 rRNA, and 1 tm RNA gene. The general features are shown in Table 1. Among the predicted CDSs, 2661 were assigned a putative function, while 1086 were predicted to encode hypothetical proteins. The average length of protein-coding genes is 913 bp. The protein-coding genes account for 89.67% of the genome sequence (Table 1 and Figure S1). 

### 3.3. Phylogenetic Analysis of Strain LQ-3

**Figure 2:**
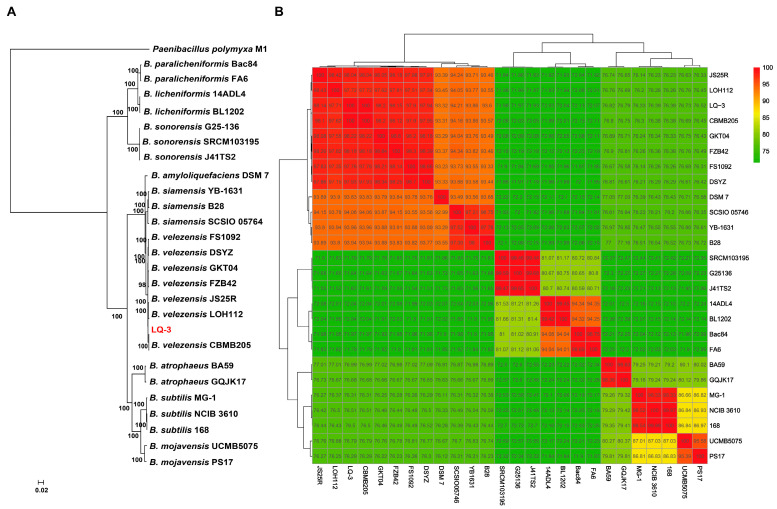
Phylogenetic analysis of *B. velezensis* LQ-3. (**A**) The ML tree of different *Bacillus* strains was generated based on 806 single-copy core genes using RAxML 8.2.10. *P. polymyxa* M1 was used as the out-group. Percent bootstrap values (from 100 replicates) are indicated at the nodes. (**B**) Heat map of ANI values among different *Bacillus* strains. The numbers represent the size of ANI values.

The construction of the phylogenetic tree for *Bacillus* genomes relied on the protein sequences of 806 core genes that were present in a single copy across all genomes. This was achieved through the use of maximum likelihood (ML) methods, and the tree was rooted by *Paenibacillus polymyxa* M1. As shown in Figure 2A, the strain LQ-3 is part of the same clade as other *B. velezensis* strains and is a sister group to *B. velezensis* CBMB205 (Figure 2A). The use of average nucleotide identity (ANI) is a highly effective method for assessing the evolutionary distance between bacterial species through digital whole-genome comparison [46]. In this study, we utilized ANI to analyze the genome relatedness of strain LQ-3. The ANI values of representative *Bacillus* strains are summarized in Figure 2B. According to the ANI values, the genome sequence of LQ-3 showed the greatest similarity with *B. velezensis* strains, with ANI values exceeding 97.15%. On the other hand, ANI values between LQ-3 and other *Bacillus* strains were lower, ranging from 71.93% to 94.21% (Figure 2B). The clustering analysis, which was based on ANI values, showed that the *B. velezensis* strains were clustered together. The heatmap analysis, which also utilized ANI values of various *Bacillus* strains, confirmed the results of the phylogenetic analysis. Above all, the strain LQ-3 was identified as *B. velezensis*.

### 3.4. Pan-genome Analysis of B. velezensis

**Figure 3:**
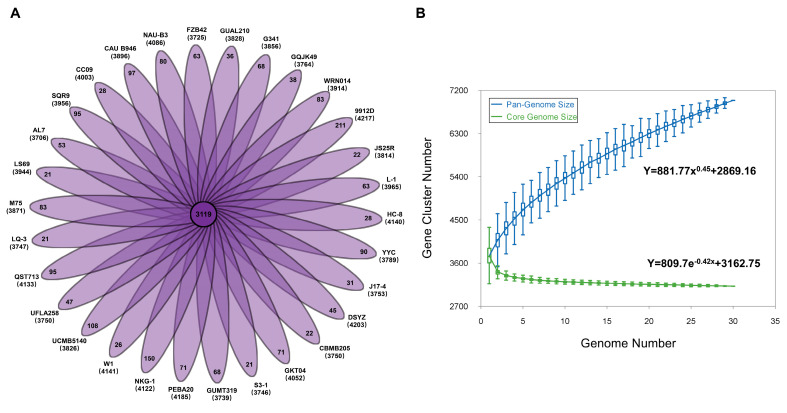

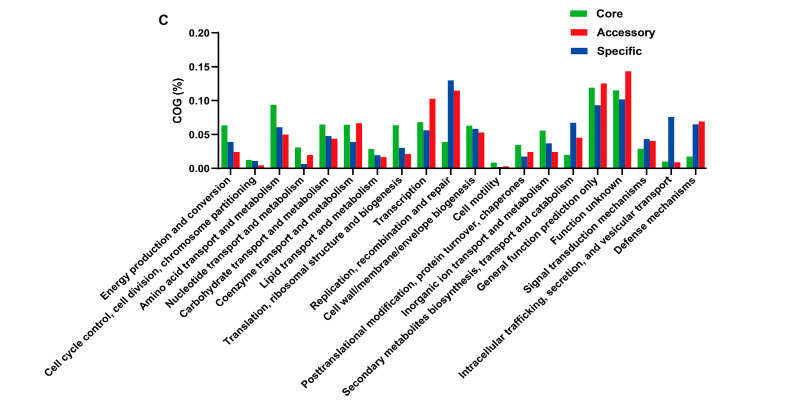
Pan-genomes of *B. velezensis* strains. (**A**) The number of specific genes for each strain of *B. velezensis*. The inner circle displays the core genomes shared between all strains. The unique genes for each strain are displayed in each of the outer circles. The number below the strain name represents the CDS of each strain. (**B**) The curves for *B. velezensis* pan-genomes and core genomes. The blue plots represent the pan-genome size of *B. velezensis* for each genome comparison, while the green plots represent the core genome size of *B. velezensis* for each genome comparison. The median values are linked to display the relationship between the number of genomes. (**C**) COG distribution of core, specific, and accessory genes present in all 30 analyzed *B. velezensis* strains.

Our analysis revealed that the pan-genome for the 30 compared *B. velezensis* strains encompasses 6989 gene families. According to the pan-genome analysis, the core genome comprises 3119 genes, while the accessory genome consists of 1999 genes, with an additional 1871 unique genes (Figure 3A). Notably, *B. velezensis* LS69, LQ-3, and S3-1 had the fewest number of specific genes, with only 21 unique genes. On the other hand, *B. velezensis* 9912D had the highest number of unique genes, with a total of 211 (Figure 3A). *B. velezensis* has a core genome that is shared by 30 strains, accounting for between 73.96% and 84.16% of the genome repertoire. The core and pan-genome sizes were calculated by extrapolating from the selected genome data. The pan-genome plot indicates that the pan-genome trend curve does not reach a plateau and appears to extend with the addition of more genomes to the analysis. Additionally, it has been observed that the generated pan-genome curves of *B. velezensis* are well-represented by the Heaps law mathematical functions: y = 881.77x^0.45^ + 2869.16, where y refers to the pan-genome size while x refers to the number of sequenced genomes. As a result, it was determined that the pan-genome is an “open” pan-genome (Figure 3B). The open pan-genome presents significant potential for uncovering new genes as more *B. velezensis* strains are sequenced [29]. Analysis of the core genome was also asymptotic, with 3119 core genes after the addition of the 30th genome. To gain a better understanding of the functional differences among *B. velezensis* strains, COG annotations were assigned to the core, dispensable, and unique genes (Figure 3C). The findings suggest that certain dispensable and unique genes in *B. velezensis* genomes may also be related to niche adaptation. The alignment conducted using the Blast Ring Image Generator (BRIG) showed that the majority of regions in the 30 *B. velezensis* genomes were conserved in comparison to the reference strain LQ-3. Several regions displayed low or no similarity, which could be due to acquisition, deletion, rearrangement, or horizontal gene transfer (Figure S2). Genes involved in c-di-GMP metabolism and regulation were primarily assigned to gene families in the signal transduction mechanism category. The DGCs, PDEs, and sensory genes were relatively conservative for *B. velezensis* strains.

### 3.5. Potential Proteins Involved in c-di-GMP Metabolism and Regulation in B. velezensis

**Figure 4:**
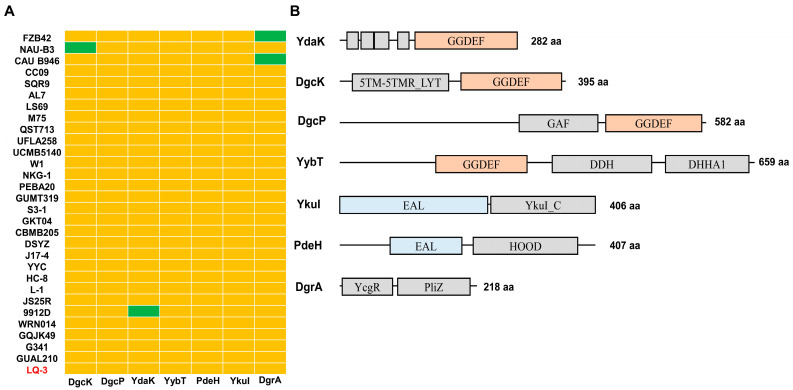
The domain analysis of seven potential c-di-GMP-related proteins in *B. velezensis* LQ-3. (**A**) Distribution of DgcK, DgcP, YdaK, YybT, PdeH, YkuI, and DgrA in *B. velezensis* group. The yellow box represents the presence of a gene within a genome, and the green box indicates the absence of a gene within a genome. (**B**) Domain composition and organization of seven c-di-GMP-related proteins in *B. velezensis* LQ-3. The orange box indicates the conserved domain of diguanylate cyclases (DGCs), the GGDEF domain. The blue box indicates the conserved domain of phosphodiesterases (PDEs), the EAL domain.

The genomes of 30 *B. velezensis* strains were downloaded from the NCBI database for further analysis. As shown in Table S2, plasmid sequences were not detected in any of the strains besides strains 9912D, NAU-B3, GKT04, and JS25R. Notably, the genome sequence of strain LQ-3 was recently released by our laboratory, and its general information is presented in Table 1 and Table S2. The average size of the genome was 4,061,011 ± 132999 bp, with an average G + C content of 46.31 ± 0.28%. The genome size varied from 3,894,709 bp in *B. velezensis* AL7 to 4,321,463 bp in *B. velezensis* DSYZ (Table S2). Additionally, we analyzed the number of proteins containing GGDEF, EAL, and PilZ domains in 30 strains of *B. velezensis*. The results showed that all strains possess genes encoding DGC enzymes in varying amounts (Figure 4A and Table S2). The strains NAU-B3 and 9912D are the only exceptions, as they contain only three proteins with the GGDEF domain, while the other 28 strains contain four. Regarding genes encoding PDE enzymes, all strains have two proteins possessing the EAL domain. In terms of proteins that contain the PilZ domain, all 28 strains, except for CAU B946 and FZB42, have at least one. Through our analysis, we discovered that LQ-3 has four proteins with GGDEF domains, including DgcK, DgcP, YdaK, and YybT, two with EAL domains, YkuI and PdeH, as well as one with a PilZ domain, DgrA (Figure 4B). This is consistent with the majority of proteins involved in c-di-GMP metabolism and regulation in *B. velezensis*.

**Figure 5:**
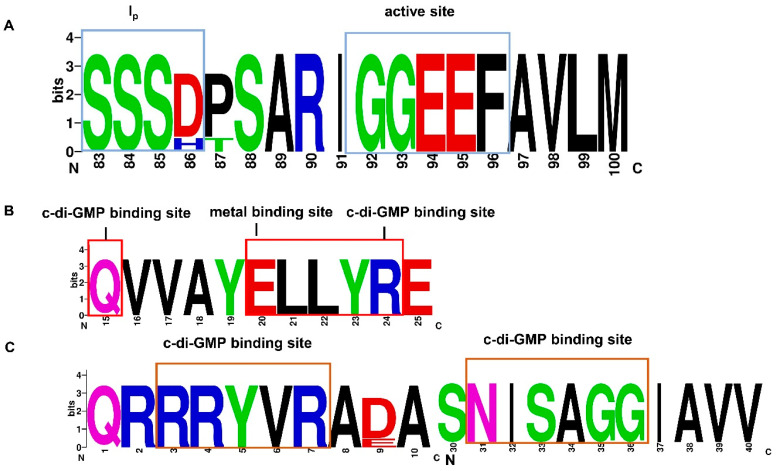
Analysis of conserved amino acid residues in the GGDEF, EAL, and PilZ domains. (**A**) Conservation analysis of amino acid residues in the GGDEF domain. The I site and catalytic site are highlighted with blue boxes. (**B**) Conservation analysis of amino acid residues in the EAL domain. The c-di-GMP binding site and metal binding site are marked with red boxes. (**C**) Conservation analysis of amino acid residues in the PilZ domain. The c-di-GMP binding site is identified by an orange box. The letters in the figure represent the abbreviations of amino acids.

We selected 16 strains of *B. velezensis* along with LQ-3 for structural domain and amino acid residue conservation analysis. Since the number of proteins containing the GGDEF and EAL domains in each strain was not unique, a uniform selection criterion was needed, selecting proteins with intact active sites and sensor domains that were essentially consistent for homologous sequence comparison and analysis. The results showed that all three domains are highly conserved in the 16 strains, with sequence homology of 98.93%, 100.00%, and 97.42%, respectively (Table S3). The conserved amino acid residues in the GGDEF domain are shown in Figure 5A, with the inhibitory site I (SXXD/H) and the active site (GGEEF) being highly conserved. The corresponding amino acid residues are Ser-Ser-Ser-Asp (SSSD), Gly-Gly-Glu-Glu-Phe (GGEEF), which are crucial for maintaining the activity of the GGDEF domain. The c-di-GMP binding site and the metal binding site in the EAL domain are also highly conserved, with the corresponding amino acid residues being Gln (Q), Arg (R), and Glu (E). In summary, the EXLXR conservative amino acid residues in the EAL domain are crucial for maintaining its activity and affect its binding to c-di-GMP (Figure 5B). The c-di-GMP binding site RxxxR and (D/N) x (S/A)xxG in the PilZ domain are also highly conserved, with arginine residues playing a key role in binding to c-di-GMP, mainly forming ionic and hydrogen bonds with the phosphate group of c-di-GMP (Figure 5C). In addition, we analyzed the active sites of seven c-di-GMP-metabolism- and regulation-related proteins in LQ-3. The results showed that the mutation of the active site in the GGDEF domain of YdaK in LQ-3 results in the loss of enzymatic activity, but it still has the ability to bind c-di-GMP. The metal binding site in the EAL domain of YkuI was mutated, which may result in a loss of enzyme activity, but it can also bind to c-di-GMP. YybT demonstrates a remarkable 93% resemblance to the extensively studied GdpP discovered in *B. subtilis*, and is expected to operate as a c-di-AMP phosphodiesterase, encompassing both GGDEF-like and DHH family domains. Consequently, we have refrained from conducting further analysis on its active sites (Figure S3). Therefore, only *dgcK*, *dgcP*, and *pdeH* among these genes might have potential metabolic enzyme activity, while *ydaK* and *ykuI* may function as c-di-GMP receptors.

### 3.6. Structural Features of the GGDEF, EAL, and PilZ Domains of B. velezensis LQ-3

**Figure 6:**
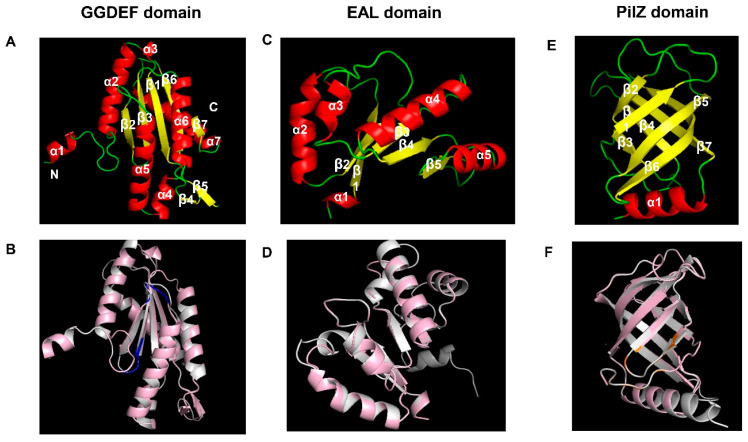
Structural features of the GGDEF, EAL, and PilZ domains in LQ-3 and their comparison with the corresponding templates. (**A**) The α helices, β strands, and loop regions of each domain are marked in red, yellow, and green, respectively. In the structural comparison, each domain is marked in pink, while the template domains are marked in white. Structural features of the GGDEF domain, using WspR (PDB id: 3I5C) from *P. aeruginosa* as the template. (**B**) The structural comparison between the GGDEF domains of LQ-3 and WspR. The SSSD and GGDEF motifs are marked in blue, corresponding to the colored boxes marking the conserved amino acid residues in the GGDEF domain. (**C**) The structural features of the EAL domain, using RmcA (PDB id: 5M3C) from *P. aeruginosa* as the template. (**D**) The structural comparison between the EAL domains of LQ-3 and RmcA. (**E**) Structural features of the PilZ domain, using MotI (PDB id: 5VX6) from *B. subtilis* as the template. (**F**) Structural comparison between the PilZ domains of LQ-3 and MotI. The RxxxR and (D/N)x(S/A)xxG motifs are marked in orange, corresponding to the colored boxes marking the conserved amino acid residues in the PilZ domain.

In this work, LQ-3 was used as a template to build a model to further understand the structural characteristics of the above domains. The GGDEF domain in WspR (Protein Data Bank (PDB) id: 3I5C) in *Pseudomonas aeruginosa* was used as the template for modeling. The results showed that the monomer of the GGDEF domain consists of 7 α helices and 7 β strands, and its arrangement from the amino terminus to the carboxy terminus is α1-α2-α3-β1-α4-α5-β2-β3-α6-β4-β5-β6-α7-β7 (Figure 6A). The Z value of this model is −1.35, lower than −4, and the sequence consistency with the template is 36.31%, indicating that the results are credible (Table S4). The RMSD value of 0.076 suggests a high degree of structural similarity between the template domain and the target domain. The I site and active site within this domain are situated in the loop regions between α5 and β2, as well as β2 and β3, respectively, as depicted in blue in Figure 6B. When homologous alignment sequences were used for EAL domain modeling, the results showed that the sequence identity with the template sequence was less than 30%, making the modeling results unreliable. Therefore, in this work, the conserved EAL structural domain sequences from the CDD analysis in NCBI’s LQ-3 were selected for modeling, and the template protein was Protein Data Bank (PDB) id: 5M3C from *Pseudomonas aeruginosa*. The results are shown in Figure 6C, where the monomer model of the structural domain consists of 5 α helices and 5 β strands alternating, with the amino acid sequence from the N-terminus to the C-terminus being α1-β1-α2-β2-α3-β3-α4-β4-α5-β5. The Z-score of the model was −2.4, with a sequence identity of 30.36% (Table S4). The RMSD value of the structural alignment was 0.084, indicating that the predicted model structure was similar to the template. The only drawback was that the important active site motif EXLXR was not reflected in the model (Figure 6D). The PilZ domain model was constructed based on the template of MotI (Protein Data Bank (PDB) id: 5VX6) from *B. subtilis*, and the results showed that the domain consisted of 7 β sheets and 1 α helix, arranged from the N-terminus to the C-terminus in the order of β1-β2-β3-α4-β4-α5-β5-β6-β7-α1 (Figure 6E). Comparing the model with MotI, the RSMD value was 0.081, indicating that the two structures were similar, and the binding site for c-di-GMP was mainly located at the N-terminus of the protein, as marked in orange in Figure 6F. The Z-score distribution of the model is shown in Table S4, and the model scores of the three domains are all above −4, indicating that the results are reliable.

### 3.7. C-di-GMP Regulates the Biocontrol-Related Phenotypes of B. velezensis LQ-3

**Figure 7:**
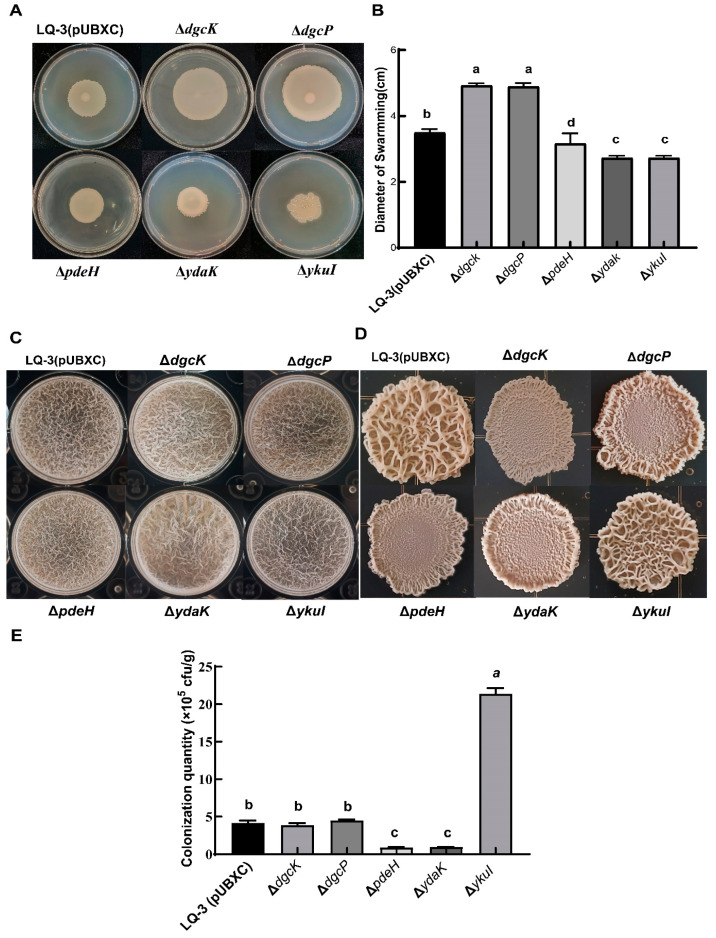
Assay of biocontrol-related phenotypes of c-di-GMP-metabolism-related proteins in LQ-3. (**A**,**B**) The swarming motility test of LQ-3 (pUBXC) and its mutant strains. (**C**) The pellicle biofilm detection of LQ-3 and corresponding mutant strains. (**D**) The colony biofilm detection of LQ-3 and corresponding mutant strains. (**E**) Colonization ability detection of LQ-3 and its mutant strains in wheat roots. The statistical analysis was performed using GraphPad Prism 8 software by one-way ANOVA with the Turkey test (*p* < 0.05). LQ-3(pUBXC): wild-type strains containing the pUBXC plasmid; *dgcK*, *dgcP*: c-di-GMP diguanylate cyclases (DGCs); *pdeH*: c-di-GMP phosphodiesterases (PDEs); *ydaK*, *ykuI*: c-di-GMP receptor.

Based on the findings, it was discovered that the genes *dgcK*, *dgcP*, and *pdeH* exhibit potential enzymatic activity, while *ydaK* and *ykuI* could potentially act as receptors for c-di-GMP. Therefore, we knocked out five genes: *dgcK*, *dgcP*, *pdeH*, *ydaK*, and *ykuI*. Subsequently, we genetically modified LQ-3 by knocking out these five genes individually. The growth of the resulting mutants did not show any significant impact on strain LQ-3 (Figure S4). Furthermore, the motility, biofilm formation, and colonization abilities of these mutants were evaluated. As shown in Figure 7, the swarming diameter of Δ*dgcK* and Δ*dgcP* was notably larger than that of LQ-3 (pUBXC), which aligns with the expected results. Surprisingly, there was no significant difference in the swarming diameter of Δ*pdeH* compared to LQ-3 (pUBXC), indicating that the PDE activity of PdeH was blocked in vivo. Mutants lacking the receptor genes, Δ*ydaK* and Δ*ykuI*, displayed reduced swarming diameters compared to LQ-3 (pUBXC), highlighting the impact of all four genes except *pdeH* on the swarming ability of LQ-3 (Figure 7A,B). However, there were no significant differences observed in the biofilm formation of pellicles for Δ*dgck*, Δ*dgcP*, Δ*pdeH*, Δ*ydaK*, and Δ*ykuI* when compared to the wild-type LQ-3 (pUBXC) (Figure 7C). Interestingly, the colony biofilm analysis revealed minor wrinkles and smoother surfaces for Δ*dgck* and Δ*dgcP* colonies in comparison to LQ-3 (pUBXC), indicating the potential diguanylate cyclase activity of DgcK and DgcP. Unexpectedly, the colony biofilm surface of Δ*pdeH* and Δ*ydaK* also showed fewer wrinkles than LQ-3 (pUBXC), suggesting that the PDE activity of PdeH in LQ-3 may be inhibited by the interaction with other proteins involved in the c-di-GMP signaling pathway. Additionally, it is speculated that YdaK may function as a c-di-GMP receptor (Figure 7D). The colonization results presented in Figure 7E indicated no significant difference in the colonization of Δ*dgcK* and Δ*dgcP* on wheat roots compared to LQ-3 (pUBXC), while the colonization of Δ*pdeH* and Δ*ydaK* decreased significantly, and that of Δ*ykuI* increased significantly, implying the involvement of *pdeH*, *ydaK*, and *ykuI* in regulating the colonization of LQ-3 on wheat roots.

## 4. Discussion

In this study, we focused on examining the c-di-GMP-metabolism- and regulation-related proteins of LQ-3 and 29 other biocontrol *B. velezensis* strains. Additionally, we investigated the role of c-di-GMP-metabolism- and regulation-related proteins in regulating the biocontrol-related traits of LQ-3. Firstly, we validated the biocontrol effectiveness of LQ-3 in managing wheat sharp eyespot through plate and greenhouse experiments. Through whole-genome sequencing and phylogenetic analysis, we confirmed LQ-3 as a *B. velezensis* strain. Additionally, the pan-genome analysis results indicated that all strains possess proteins associated with c-di-GMP metabolism and regulation, specifically belonging to the signal transduction family. Furthermore, the quantity and activity sites of c-di-GMP metabolism and conserved regulatory protein domains were found to be consistent among different bacterial strains. Finally, we examined the functions of c-di-GMP-metabolism-related proteins in regulating motility, biofilm formation, and colonization in the roots of wheat in LQ-3. and the regulation of the above biocontrol phenotypes by PdeH may be mediated through its interaction with other proteins. Overall, our findings provide preliminary insights into the c-di-GMP signaling pathway in biocontrol strain LQ-3.

The application of biocontrol agents, such as *Bacillus*, for the control of plant diseases has emerged as a research focus in recent years [47,48]. In this study, we conducted plate antagonistic experiments using the fermentation broth of LQ-3, which indicates that the strain LQ-3 is a potential biocontrol strain (Figure 1). This study further confirmed that *B. velezensis* LQ-3 is effective in controlling wheat sharp eyespot through potting experiments (Figure 1). However, it is important to note that the effectiveness of LQ-3 against wheat sharp eyespot was only assessed in greenhouse conditions, which imposes certain limitations. To obtain a more comprehensive understanding, field experiments are still necessary. As previously mentioned, the efficacy of biocontrol agents in the field can be influenced by environmental conditions and their intrinsic genetic factors [5,6,7]. The complex field environment is susceptible to various factors, including wind and rainfall, which can impact the effectiveness of these strains. Hence, the research aimed at enhancing the colonization ability of biocontrol strains in crop roots and their environmental tolerance ought to be sustained.

C-di-GMP can regulate the pathogenic activity, motility, and biofilm formation of biocontrol *Bacillus* [22,49]. In general, receptors of c-di-GMP modulate their physiological activities in response to varying environmental conditions by sensing the concentration changes in c-di-GMP. Therefore, the types and quantities of c-di-GMP metabolic enzymes are crucial. The quantities of c-di-GMP metabolism and regulatory proteins vary among different bacteria. For instance, *Vibrio cholerae* has over 60 proteins with GGDEF-EAL domains, while *Xanthomonas campestris* has at least 37 proteins with HD-GYP, GGDEF, and/or EAL domains. However, *Mycobacterium smegmatis* only has one c-di-GMP metabolic enzyme, and *B. subtilis* NCIB 3610 has six [23,24,50,51,52]. In this study, it was found that the majority of the 30 *B. velezensis* strains examined had six proteins with GGDEF/EAL domains, and one PilZ domain protein. These findings are consistent with those of *B. subtilis*, with the exception of the absence of a protein known as YkoW, which contains both GGDEF and EAL domains [23]. *B. velezensis* does not have any proteins with the HD-GYP domain. Similarly, all *Bacillus* strains lack HD-GYP domain proteins, except for the animal pathogenic *B. anthracis* [53]. The specific reasons for this exception need further investigation. It has been suggested in the literature that the variation in the amounts of GGDEF/EAL/HD-GYP domain proteins in different bacteria could be attributed to environmental factors and intracellular c-di-GMP signaling pathways [9,54,55]. Additionally, certain bacteria possess proteins containing GGDEF and EAL domains, which typically serve a singular function in either synthesis or degradation due to the loss of one domain. However, a subset of these proteins exhibited dual functionality in c-di-GMP metabolism. In *Dickeya dadantii*, the c-di-GMP metabolic enzyme EcpB has both GGDEF and EAL domains but primarily exhibits PDE activity. On the other hand, the c-di-GMP metabolic enzyme ScrC in *Vibrio parahaemolyticus* has dual functions in both synthesis and degradation [9,56].

The function of a protein is closely related to its structure. In this study, we analyzed the active sites in these conserved protein domains and constructed models to explore the functions of c-di-GMP metabolism and regulatory proteins in LQ-3. The results showed that the active sites of GGDEF, EAL, and PilZ domains were highly conserved in different strains. It has been reported that the GGDEF domain topology in *Bacillus menisulans* is α0-β1-α1-α2-β 3-α3-β4-β5-β6-α4-β7, which is similar to LQ-3 [57], and their active sites are located between β2 and β3. The difference is that the amino end of the GGDEF domain in LQ-3 has two small alpha helical structures, indicating that the GGDEF domain remains highly conserved in both Gram-positive and Gram-negative bacteria, and its function has not changed significantly.

C-di-GMP can regulate various biocontrol-related functions of *Bacillus*, including motility, biofilm formation, and colonization. To date, only PG12 and SQR9 have been studied for the c-di-GMP signaling pathway in *B. velezensis*. The swarming motility of Δ*dgcK* and Δ*dgcP* was significantly higher than that of the wild type in PG12, while Δ*pdeH* exhibited the opposite phenotype [22]. In SQR9, the swarming motility of Δ*dgcK* was significantly higher than that of the wild type, while Δ*dgcP*, Δ*ydaK*, and Δ*ykuI* showed no significant difference, and Δ*pdeH* exhibited significantly lower swarming motility compared with the wild type [21]. In this study, the swarming motilities of Δ*dgcK*, Δ*dgcP*, and Δ*pdeH* were only consistent with those in PG12, while Δ*ydaK* and Δ*ykuI* showed reduced swarming motility compared to LQ-3(pUBXC), which was different from SQR9. Moreover, the swarming motility of Δ*pdeH* did not exhibit significant variance compared to the wild type. This phenotypic alteration diverged from both PG12 and SQR9, suggesting the PDE activity of PdeH in LQ-3 may be blocked by the interaction with other proteins involved in the c-di-GMP signaling pathway. It is speculated that *ydaK* and *ykuI* act as receptors for c-di-GMP to regulate the swarming motility of LQ-3. For biofilm formation, there is no difference in the pellicle biofilm morphology of Δ*dgcK*, Δ*dgcP*, Δ*pdeH*, Δ*ydaK*, and Δ*ykuI* compared to LQ-3(pUBXC) in PG12 [22]. However, the results of the study in SQR9 indicate that Δ*dgcP* and Δ*ykuI* had significantly reduced biofilm formation, and their colonization in wheat roots was also reduced accordingly. In LQ-3, the morphological changes in pellicle biofilm in the above mutants were similar to those in PG12, while the colony biofilm morphology was different from that in SQR9. Our study results showed that the deletion of *dgcK*, *dgcP*, *pdeH*, and *ydaK* separately led to significantly reduced colony biofilm formation. Moreover, the colonization of Δ*pdeH* and Δ*ydaK* in wheat roots also decreased. It is speculated that the weakened colonization of Δ*pdeH* and Δ*ydaK* in wheat roots is due to their weakened biofilm formation. And the colonization of Δ*ykuI* in wheat roots significantly increased compared to LQ-3 (pUBXC); however, its biofilm morphology seems to have no difference compared with the wild type, supposing that it functions through other signaling pathways rather than as a c-di-GMP receptor. These study results suggest that DgcK and DgcP function as diguanylate cyclases, and hence, the intracellular c-di-GMP concentration of Δ*dgck* and Δ*dgcP* decreased, resulting in weakened biofilm formation. While the intracellular c-di-GMP concentration of Δ*pdeH* should increase, and its ability to form biofilm should be enhanced or remain unchanged due to the concentration not reaching the threshold that causes changes in biofilm formation, this study showed the opposite phenotypes, speculating that the PDE activity of PdeH was blocked in vivo. PdeH directly regulates the formation of LQ-3 swarming motility and colony biofilm through interacting proteins involved in the c-di-GMP signaling pathway, suggesting that a localized signaling pathway exists in *B. velezensis* LQ-3.

Two hypothetical models have been proposed for the c-di-GMP signaling pathway in bacteria: the global signaling pathway and the local signaling pathway [58,59]. Recent research has demonstrated that *Salmonella enterica* possesses two c-di-GMP receptors, YcgR and BcsA, which exhibit distinct affinities towards c-di-GMP. YcgR, which binds to c-di-GMP, is responsible for regulating bacterial cell mobility when intracellular levels of c-di-GMP are low. Conversely, BcsA binds to c-di-GMP when its concentration rises above a certain threshold, regulating the production of cellulose. The two receptors function in tandem to regulate the phenotype of the bacteria [60]. The global c-di-GMP signaling pathway model was initially proposed based on this phenomenon. However, through recent research into the c-di-GMP signaling pathway, it has been discovered that the metabolic enzymes DGCs/PDEs are co-localized with c-di-GMP receptors, leading to the proposal of a local signaling pathway model. In *Escherichia coli*, biofilm formation is regulated by a local c-di-GMP signaling pool. This pool is controlled by a pair of DGC/PDE enzymes (YdaM and YciR), which are in turn regulated by another pair of DGC/PDE enzymes (YegE and YhjH). The downstream gene expression related to biofilm formation is impacted by the activity of these enzymes. YciR serves a dual function as a degradation enzyme for c-di-GMP and a receptor, referred to as a ‘trigger enzyme’. Additionally, YdaM and YciR are present in a complex with the downstream protein MlrA. This signaling pathway regulates the metabolism enzymes, receptors, and downstream regulators of c-di-GMP through protein interactions to control the formation of bacterial biofilms [58].

## 5. Conclusions

*B. velezensis* LQ-3 is a potent biocontrol strain for combating wheat sharp eyespot disease caused by *R. cerealis*. The strain exhibits biofilm-forming and motility capabilities. Our study revealed the presence of seven proteins in LQ-3, including four proteins with GGDEF domains, two proteins with EAL domains, and one protein with a PilZ domain. The proteins analyzed in our study were found to belong to the signal transduction family, as determined by pan-genome analysis. Our results indicated a high degree of conservation in the active sites of GGDEF, EAL, and PilZ domains across different *B. velezensis* strains, providing preliminary insights into the structural characteristics of the above domains in LQ-3. DgcK, DgcP, PdeH, YdaK, and YkuI all play a role in regulating the motility, biofilm formation, and colonization of LQ-3 in wheat roots to varying degrees. These findings preliminarily clarify the c-di-GMP signaling pathway associated with disease resistance in LQ-3. Further studies are required to elucidate the specific role of c-di-GMP metabolism and regulatory proteins in disease prevention. Additionally, it is necessary to determine whether these proteins confer resistance to wheat sharp eyespot by regulating the biocontrol-related traits of LQ-3.

## Figures and Tables

**Table 1:** The general features of the genome of *B. velezensis* LQ-3.

Attribute	LQ-3
Genome size (bp)	3,929,792
G + C (%)	46.50
Protein-coding genes	3747
rRNA	27
tRNA	86
Gene total length (bp)	3,523,857
Average length of protein-coding genes (bp)	913

## Data Availability

The datasets analyzed during the current study are available in the NCBI GenBank under accession number CP120637.

## Supplementary Materials

The following supporting information can be downloaded at https://www.mdpi.com/article/10.3390/microorganisms12081724/s1, Figure S1: Genome map of *B. velezensis* LQ-3. Figure S2: Circular representation of the *B. velezensis* LQ-3 genome and comparative genomics analysis with other *B. velezensis* strains generated by BRIG. Figure S3: Analysis of the active sites of the structural domains of c-di-GMP-metabolism-related proteins in *B. velezensis* LQ-3. Figure S4: Growth rate determination of LQ-3 (pUBXC) and its corresponding mutant strains. Table S1: The list of bacterial strains having potential activities in the control of *R. cerealis*. Table S2: The general genome features and predicted modular signaling proteins involved in c-di-GMP metabolism in all 30 analyzed *B. velezensis* genomes. Table S3: *B. velezensis* utilized for homologous sequence comparison and analysis of the GGDEF domain, EAL domain, and PilZ domain. Table S4: QMEAN Z-score of predicted GGDEF, EAL, and PilZ domain models and their sequence consistency with the templates. Table S5: Strains and plasmids used in this study. Table S6: Primers used in this study.
