# Supplementary material for: Comparative Genomic and Functional Analysis of c-di-GMP Metabolism and Regulatory Proteins in Bacillus velezensis LQ-3
Source: Microorganisms. 2024 Aug 21;12(8):1724. doi: 10.3390/microorganisms12081724 (PMC11357230; doi:10.3390/microorganisms12081724)
Supplement: Supplementary file 1 [file microorganisms-12-01724-s001.zip › supplementary materials/FigureS1-S4.pdf]

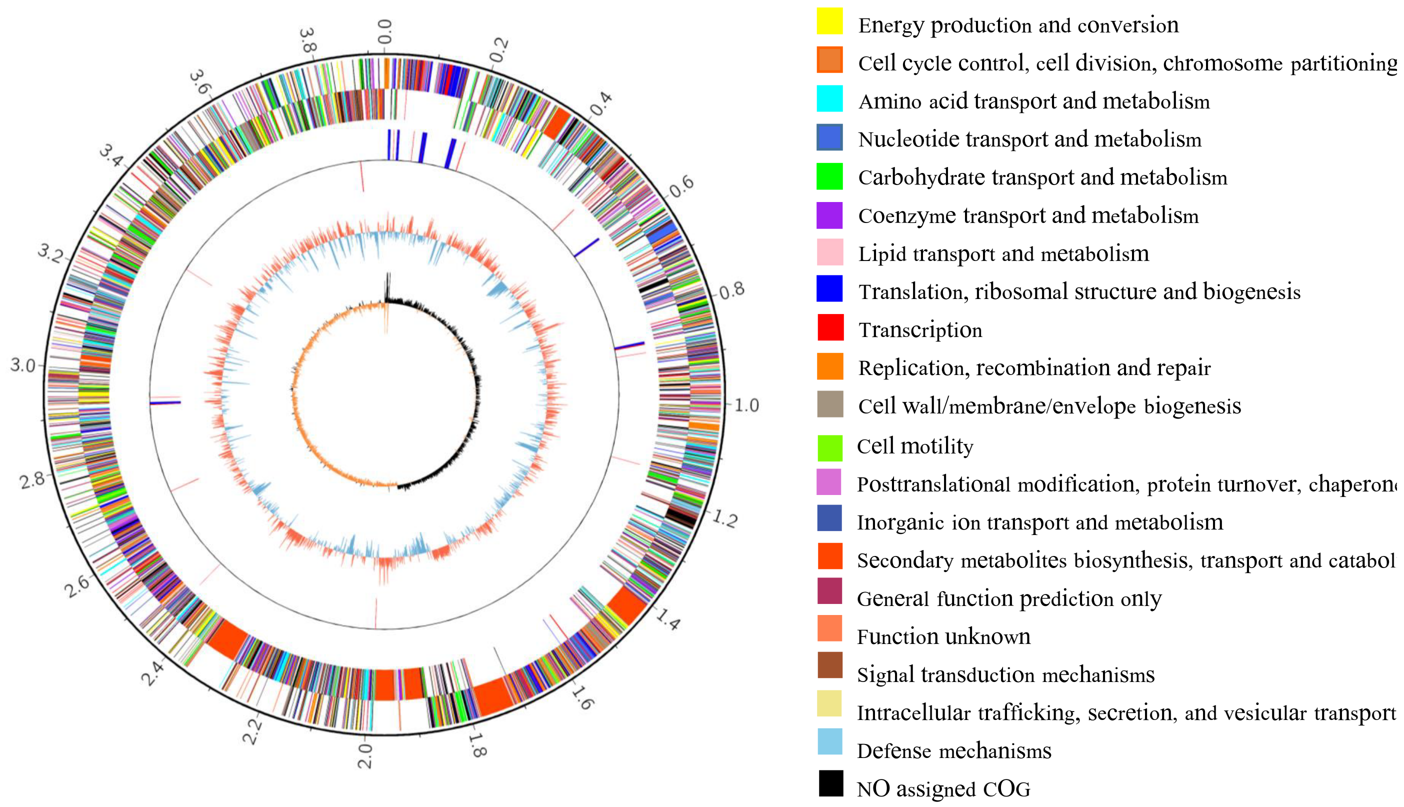

Figure S1 Genome map of *B. velezensis* LQ-3. Each ring of the circle represents a different genome information: (1) scale marks (unit: Mb), (2, 3) protein-coding genes on the forward and reverse strands, respectively (color-coded by the functional categories), (4, 5) rRNA (blue) and tRNA (red) on the forward and reverse strands, respectively, (6) GC content (positive: red; negative: blue), and (7) GC skew (above average: aquamarine; below average: orange).

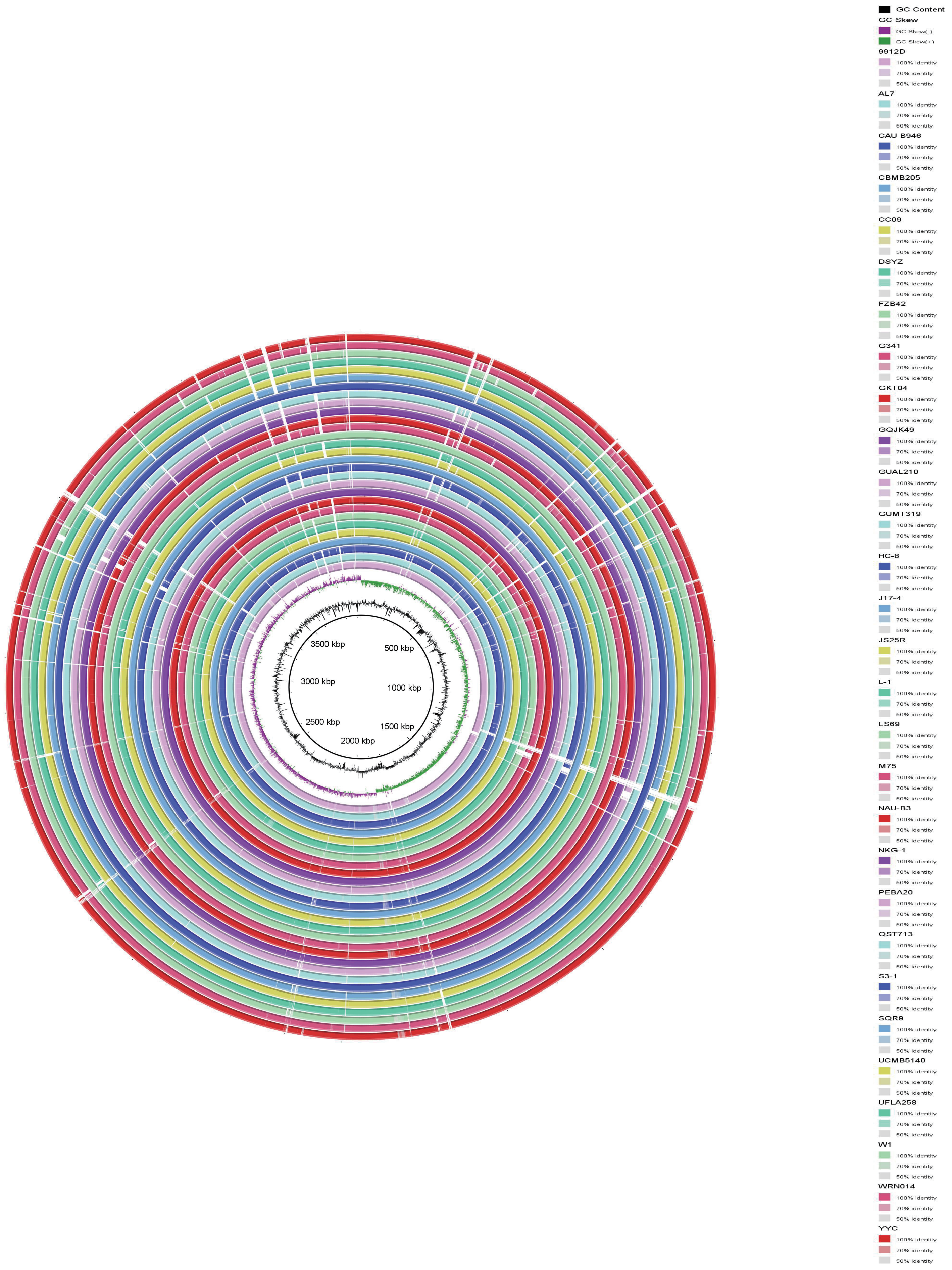

Figure S2 Circular representation of the *B. velezensis* LQ-3 genome and comparative genomics analysis with other *B. velezensis* strains generated by BRIG.

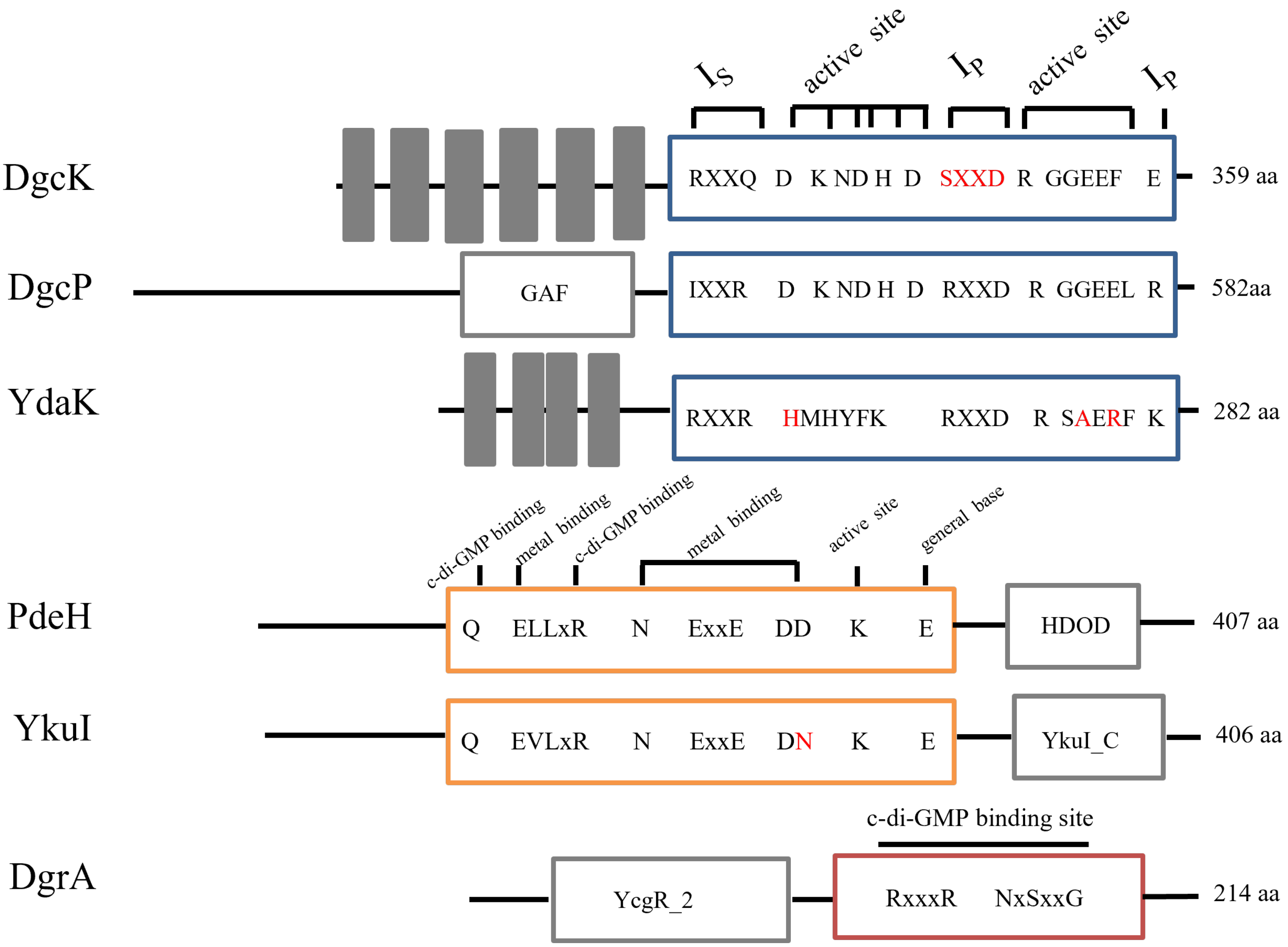

Figure S3 Analysis of the active sites of the structural domains of c-di-GMP metabolism-related proteins in *B. velezensis* LQ-3.

DgcK, DgcP: c-di-GMP diguanylate cyclases (DGCs)

PdeH: c-di- GMP phosphodiesterases (PDEs)

DgrA, YdaK, YkuI: c-di-GMP receptor

I_P: the primary inhibitory active sites

I_s: secondary inhibitory active sites

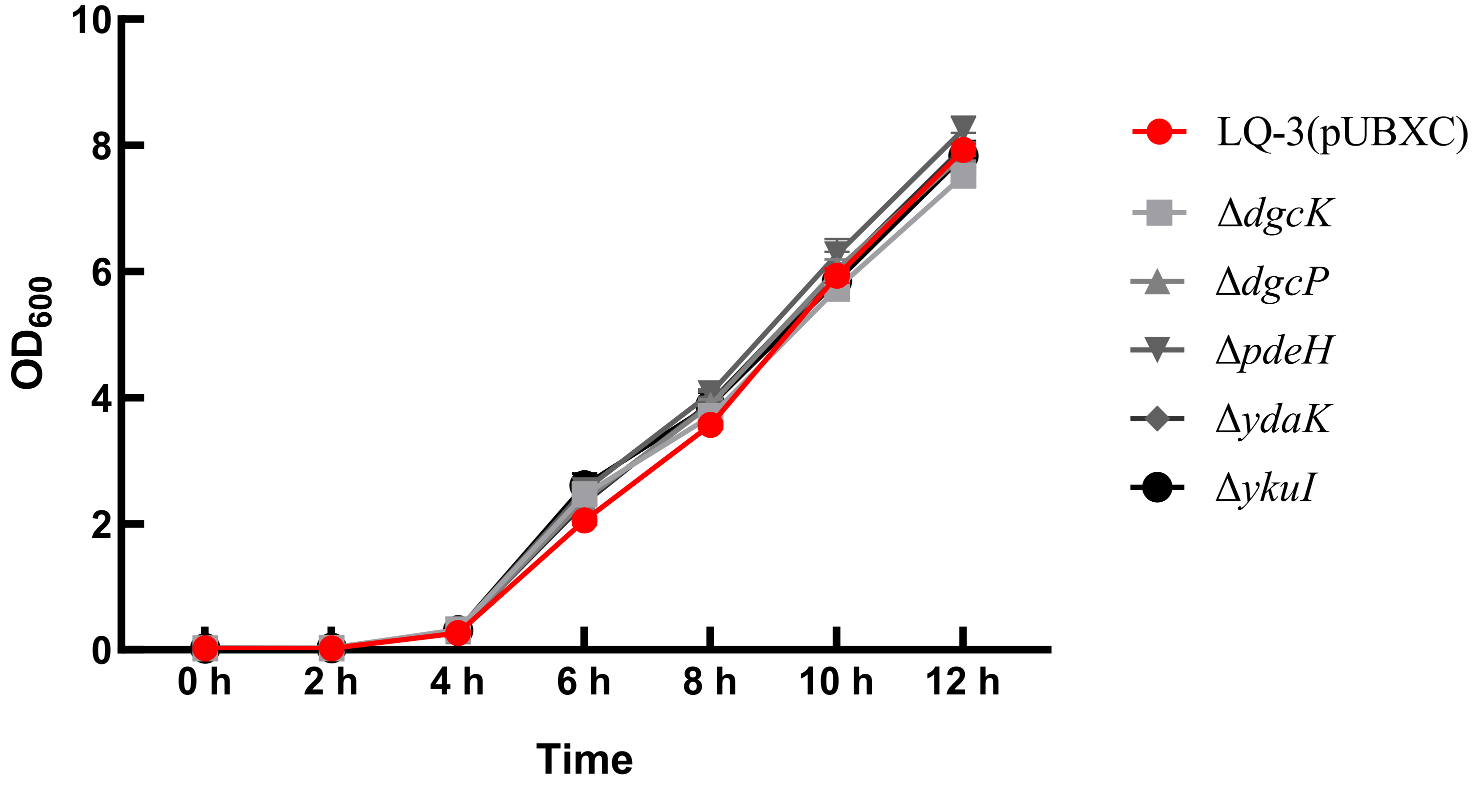

Figure S4 Growth rate determination of LQ-3 (pUBXC) and its corresponding mutant strains.

LQ-3(pUBXC)： The LQ-3 strain containing the pUBXC plasmid.

dgcK, *dgcP*: c-di-GMP diguanylate cyclases (DGCs)

pdeH: c-di- GMP phosphodiesterases (PDEs)

ydaK, *ykuI*: c-di-GMP receptor

"Δ" refers to a mutation.
